# Supplementary material for: scRNA-seq reveals persistent aberrant differentiation of nasal epithelium driven by TNFα and TGFβ in post-COVID syndrome
Source: Nat Commun. 2025 Oct 28;16:9494. doi: 10.1038/s41467-025-64778-0 (PMC12569062; doi:10.1038/s41467-025-64778-0)
Supplement: Supplementary file 1 — Supplementary Information [file 41467_2025_64778_MOESM1_ESM.docx]

**Supplementary File**

**Material and Methods**

**NAPKON samples population selection breakdown**

We obtained nasal biopsies from the anterior and medial head of the middle turbinate under endoscopic guidance, from 33 PCS patients (Figure 1). This standard procedure uses a curette to scrape out well-defined biopsies containing both epithelial and immune cells. Patients were registered in the NAPKON-POP cohort and had provided written and informed consent before biopsy collection, aligning with ethical approval. The criteria for patient inclusion were (i) polymerase chain reaction confirmed SARS-CoV-2 infection and persistence of COVID-19 symptoms for more than three months, (ii) post-acute infection symptom development, (iii) a worsening of pre-existing comorbidities, and (iv) written and informed consent before biopsy collection, aligning with ethical approval. A breakdown of the number of samples selected from the NAPKON cohort is provided in Supplementary Figure S1.

**Single-cell library prep and sequencing parameters**

Single-cell sequencing was performed in collaboration with the Singleron Company, Cologne, Germany, whose workflow allows storage of tissue specimens up to 72 hours before processing and library preparation, ensuring high sample integrity. Nasal curettage samples were collected from study participants at the University Hospital Schleswig-Holstein (UKSH), Campus Kiel, Germany, during routine patient visits. After isolation cell suspensions were shipped overnight at 4°C to Singleron Labs in Cologne, Germany. Cell viability was assessed at Singleron Labs, and samples with viability exceeding 80% were subsequently processed into single-cell suspensions using the sCelLiVE Tissue Dissociation Solution (#1190062, Singleron). Library preparation was carried out using the GEXSCOPE Single Cell RNA Library Kit on a microwell chip (SCOPE-chip) with barcoded beads. Cells were subsequently lysed and the beads with attached poly-A tailed transcripts extracted. The captured mRNA was then reverse transcribed into cDNA, amplified, fragmented, and then ligated with sequencing adapters. The finished library was paired-end sequenced on an Illumina NovaSeq.

**Single-cell RNA-Seq analysis pipeline**

Raw gene expression matrices were generated for each sample by a custom pipeline combining *‘kallisto’* (v.0.46.1) and *‘bustools’* (v.0.46.1) using GRCh38 as human reference. The output-filtered gene expression matrices were analyzed in 'R' (v.4.2.1), where empty droplets and doublets were removed for each sample using the packages 'DropletUtils' (v.1.8.0) and ‘doubletFinder’^1^, and further analysis was performed using the 'Seurat' (v.4.3) package. Subsequently, cells were detected by ranking cell barcodes according to their number of unique molecular identifiers (UMIs) captured using the barcodeRanks function. Low-ranked cells from this process were labelled as false positives and were discarded, yielding 218x10^6^ unique reads with an average of 4000 reads per cell. The criteria for filtering the dataset were as follows: (i) genes expressed in more than three cells; (ii) cells expressing more than 200 genes; (iii) low-quality cells were removed if they included more than 25% UMIs from the mitochondrial genome. Mitochondrial content can vary between humans and mice based on scRNA-seq technology. Mercer et al.^2^ found mtDNA% ranges from 5% in low-energy tissues to 30% in high-energy tissues. We set the filter to 25% after calculating mitochondrial content for each sample. Gene expression matrices were normalized by the NormalizeData function, and 2,000 features with high cell-to-cell variation were calculated using the FindVariableFeatures function. To correct for batch effects, we used integration methods selected according to the structure and complexity of each dataset. For the nasal swab biopsy samples, which exhibited substantial heterogeneity and batch variation, we applied Seurat’s canonical correlation analysis (CCA)-based workflow using the FindIntegrationAnchors and IntegrateData functions. In contrast, for the more homogeneous ALI culture validation dataset, we used Harmony³, a fast and scalable method that effectively removes technical artifacts while preserving biological variation. Each integration method was applied independently to its respective dataset based on its suitability for the data type and study design^4^. The dimensionality of the integrated dataset was reduced to 100 principal components of the linearly scaled data using the ScaleData and RunPCA functions, respectively. Finally, we clustered cells using the FindNeighbors and FindClusters functions and performed nonlinear dimensionality reduction by uniform manifold approximation and projection for dimension reduction (UMAP) with the RunUMAP function, using 30 dimensions for all approaches. The FindAllMarkers function in Seurat was used to find markers for each unique cluster. Clusters were identified and annotated based on expression of canonical markers for epithelial cell types (Source data, Supplementary Table S2). A manual expert annotation, considered the gold standard for cell annotation^5^, was performed. In this process, gene expression by cells was examined using various resources (Human Lung Atlas^6, 7^). Although expert manual annotation is regarded as the gold standard for cell annotation^5^.

**Viral RNA-seq analysis**

*Fastq reads were aligned with STAR 2.7.3 to the SARS-CoV-2 reference genome (Assembly ASM985889v3), revealing only 228 cells with successful alignment out of 218×10^6 unique reads, indicating a negligible viral load of 0.0001078%.*

**Determination of cell abundance**

We used the ‘scProportion Test’ from the R library scProportionTest’ to quantify differences in cell abundance between clusters from two scRNA-seq samples. A permutation test was used to calculate a statistical p-value for each cluster, and a confidence interval for the magnitude difference was returned via bootstrapping^8^.

**Differential abundance algorithm**

A permutation test was used to calculate a statistical p-value for each cluster, and a confidence interval for the magnitude difference was returned via bootstrapping^8^. Differential abundance analysis was also applied via the algorithm DAseq (version 1.0.0) input. This involved the union of data from moderate and severe PCS after dimension reduction. In the initial step, DA-seq calculates a score vector for each cell, reflecting the relative prevalence of cells from different biological states in its neighborhood. This process incorporates neighborhood sizes at multiple scales, providing a comprehensive view of differential abundance for each cell. This is done to reduce the variance error and resolve the selection of the optimal k value (which is usually unknown). The multiscale score vectors are amalgamated into a unified measure of differential abundance using a *logistic regression classifier* trained to predict the biological state of each cell based on its score vector entries. The resulting prediction probabilities are then transformed into a differential abundance measure, indicating the extent to which a cell's neighbourhood is dominated by cells from one biological state. A *permutation test* to determine which of the DA measures computed for each cell in step 2 is statistically significant (threshold set to distinguish which cells are differentially abundant and which are not) (Supplementary Figure S5 A+B). DA-seq clusters cells with DA measures surpassing or falling below a predefined threshold into localized regions based on their gene expression profiles. These regions represent cell subpopulations exhibiting significant differences in abundance between biological states. Each identified DA subpopulation is assigned a DA score and accompanied by a P-value to evaluate reproducibility, given sufficient biological replicates in both states. The algorithm selects genes that differentiate a DA subpopulation from other cells in the dataset or from its immediate neighborhood. To achieve this, it utilizes a recently developed feature selection method called stochastic gates (STG) to identify the minimum number of genes necessary to distinguish a DA subpopulation. Additionally, standard differential expression methods are employed to complement this selection process.

**TriNetX cohort selection and analysis**

We retrieved a case and control cohort for post-COVID symptoms according to the International Statistical Classification of Diseases and Related Health Problems (ICD-10) codes from the TriNetX Global Collaborative Network^9^. This network provides access to electronic medical records from 107 healthcare organizations (HCOs). This retrospective study is exempt from informed consent. data reviewed is a secondary analysis of existing data, does not involve intervention or interaction with human subjects, and is de-identified per the de-identification standard defined in Section §164.514(a) of the HIPAA Privacy Rule. The process by which the data is de-identified is attested to through a formal determination by a qualified expert as defined in Section §164.514(b)(1) of the HIPAA Privacy Rule. A total of 77 providers responded with a total of 52,833 samples having a diagnosis of post-COVID syndrome disease, unspecified (U09.9; ICD-10-CM) and must not have COVID-19 (U07.1; ICD-10-CM) and COVID-19 virus not identified (U07.2; ICD-10- CM). As controls, we selected people presenting for general examination with no complaints “Encounter for general examination without complaint, suspected or reported diagnosis” (ICD10CM:Z00) and had neither COVID-19 nor post-COVID diagnosis, resulting in a total of 15,797,934 patients from 105 HCOs. To balance the cohorts, propensity score matching was performed based on age, age at index, and gender to address confounding factors. The selection process is represented in Supplementary Figure S17A. A total of 69 diseases related to respiratory and nasal complications were selected according to their ICS-10 codes and their odds and hazard ratios between cases and controls were calculated according to the "Compare Outcomes" function of the TriNetX online interface (Supplementary Table S4).

**Cell interaction Analysis**

To identify and visualize the cell cross-talk among cells or between clusters, the R package ‘CellChat’ (v.1) was used according to the developer’s vignette [<https://github.com/sqjin/CellChat>]^10^. ‘CellChat’ leverages known ligand-receptor pairings to determine the probability of signaling interactions at a single-cell resolution. Each pathway of the network is determined by summing all interaction strengths of the ligand-receptor pairs, via a quantification of their respective expression levels on different cell types^10^.

**Differential Pathway Enrichment Analysis – PROGENy**

To identify molecular pathways that are differentially regulated between moderate and severe PCS patients, the PROGENy database was used^11^. The PROGENy database, incorporating empirical activity weights from perturbation experiments for perturbation response genes, was used to estimate the activity of 14 signaling pathways. We applied the `progeny()` function to the gene expression matrix, setting the parameters for organism to "Human" and selecting the top 500 pathway-responsive genes for each pathway. Pathway scores were computed at the single-cell level, scaled using Seurat’s `ScaleData()` function, and converted into a long-format data frame. The pathway activity is statistically tested between moderate and severe PCS groups by incorporating a linear model^11^.

**Differential Gene Expression and Gene Set Enrichment**

Differential gene expression analysis of the single-cell data was performed per cell cluster and PCS group using the MAST method (Model-based Analysis of Single-cell Transcriptomics), as implemented in the FindMarkers() function from the Seurat R package . MAST uses a hurdle model tailored for single-cell RNA-seq data, allowing it to model both the discrete and continuous components of gene expression and incorporate sample-level covariates to reduce pseudoreplication bias^12^**.** Benchmarking analyses revealed that MAST ranked among the best-performing methods under conditions of moderate sequencing depth and batch effects, which closely reflected the characteristics of our dataset. Given our sample size, we therefore identified MAST as the most robust approach for this analysis ^13^. Gene set enrichment was calculated using a non-parametric Wilcoxon Mann-Whitney test on the log fold changes between the cell groups as implemented in R library gage (v. 2.52)^14^.To address also pseudoreplication biases we additionally performed a pseudobulk DE analysis using Seurats’s *AggregateExpression* function and DESeq2, followed by GSEA with a Wilcoxon Mann-Whitney test on log fold changes (see Reviewer Figure S1). This independent approach confirmed the enrichment of TNFα, NF-κB, and EMT pathways in severe PCS patients (Supplementary Figure S18; page 8, lines 238–242), supporting the robustness of our findings .

**Pseudotime**

Single-cell pseudotime trajectories were constructed using Monocle (version 2.6.4)^15^. Briefly, we first selected a set of ordering genes that showed differential expression between the clusters. Subsequently, Monocle then uses reversed graph embedding, a machine learning technique, to generate a parsimonious principal graph and then reduces the given high-dimensional expression profiles to a low-dimensional space. Individual cells are projected onto this space and ordered along trajectories that are connected by branching points, which correspond to cell fate decisions. The resulting graph structure was then characterized for network centrality as a proxy of wild-type or impaired cell differentiation.

**Deconvolution**

We used Dampened weighted least squares (DWLS) as the estimation method for gene expression deconvolution. With DWLS [https://github.com/dtsoucas/DWLS], the expression levels of marker genes are used to estimate a cell type-specific density distribution for each marker gene in the dataset^16^. These marker genes are derived from genes that are highly expressed in each cell type in the single-cell dataset. After this estimation, DWLS uses adjusted least square regression to match the different cell types in the single-cell dataset to the bulk data. The output will then show the proportion of cell types that match the expression of the bulk dataset.

**Members of ALLIANCE Study Group**

Mira Bürk^a^ MA; Sybille Contento^a^ MD; Markus Ege^tc^ MD; Silvia Gschwendtner ^v^ Ph.D.; Alexander Hose^a^, Ph.D., MA, MPH; Sabina Illi^c^ Dr., Dipl.-Stat., MPH; Constanze Jakwerth ^X^ Ph.D.; Lena Lagally^a^ MSc; Nicole Maison^a,c^ MD; Jimmy Omony^c^ Ph.D.; Bianca Schaub^ay^ MD; Michael Schloter ^v^ Ph.D.; Carsten Schmidt-Weber ^X^ Ph.D.; Lena Ullemeyer^a^ MSc; Erika von Mutius^a,c^ MD MSc; Esther Zeitlmann^a^ Dipl.oec.troph; Mustafa Abdo^k^ MD; Thomas Bahmer^k^ MD; Heike Biller^k^ MD; Constantin Blanke-Roeser^k^ MD; Folke Brinkmann^d^ MD; Karoline I. Gaede^m^ PhD; Wiebke Hagedorn^d^ MD; Nikolas Jacobs^d^ MD; Anne-Marie Kirsten^l^ MD; Inke R. König^e^ PhD; Matthias V Kopp^b,d^ MD; Lea Kronziel^e^ MSc; Gyde Nissen^d^ MD; Wasifa Nurieva^d^ PhD; Frauke Pedersen^k^ PhD; Loana Penner^d^ MD; Klaus F. Rabe^k^ MD PhD; Isabell Ricklefs^d^ MD; Silke Szymczak^e^ PhD; Vera Veith^k^ PhD; Benjamin Waschki^k^ MD; Henrik Watz^l^ MD; Markus Weckmann^d,u^ PhD; Christopher Wirks^k^ MD; Marie Bickes^f^ MD; Mifflin-Rae Calvero^n^ MSc; David S. DeLuca^n^ PhD; Anna-Maria Dittrich^f^ MD; Svenja Gaedcke^n^ MSc; Ruth Grychtol^f^ MD; Anika Habener^f^ Dipl.-Biol.; Gesine Hansen^f^ MD; Christine Happle^f^ MD; Adan Chari Jirmo^f^ PhD; Bin Liu^n^ MSc; Lennart Riemann^f^ MD; Svenja Foth^h^ MD; Inga Jerrentrup^h^ MD; Harald Renz^o^ MD; Chrysanthi Skevaki^o^ MD; Stefanie Weber^h^ MD; Miguel A. Alejandre Alcazar^p,q,r,s^ MD, PhD; Lena Keufken^j^ PhD; Silke van Koningsbruggen-Rietschel^j^ MD; Ernst Rietschel^j^ MD; Tobias Trojan^j^ MD; Jan-Christoph Thomassen^j^ MD

**Affiliations**

**a** Department of Paediatric Allergology, Dr von Hauner Children’s Hospital, Ludwig Maximilians University, Munich, Germany, and Comprehensive Pneumology Center, Munich (CPC-M), Germany; German Center for Lung Research (DZL); **b** Department of Paediatric Respiratory Medicine, Inselspital, University Children's Hospital
of Bern, University of Bern, Bern, Switzerland; **c** Institut für Asthma- und Allergieprävention (IAP), Helmholtz Zentrum Munich, Deutsches Forschungszentrum für Gesundheit und Umwelt (GmbH), Munich, Germany; **d** University Children’s Hospital, Luebeck, Germany, and Airway Research Center North (ARCN), Germany; German Center for Lung Research (DZL); **e** Institute for Medical Biometry and Statistics, University Luebeck, University Medical
Centre Schleswig-Holstein, Campus Luebeck, Germany, and Airway Research Center North (ARCN), Germany; German Center for Lung Research (DZL); **f** Department of Paediatric Pneumology, Allergology and Neonatology, Hannover Medical School, Hannover, Germany, and Biomedical Research in Endstage and Obstructive Lung Disease Hannover (BREATH), Germany; German Center for Lung Research (DZL); **g** Department of Paediatric Pneumology, University Children’s Hospital, Ruhr-University
Bochum, Bochum, Germany; **h** University Children’s Hospital Marburg, University of Marburg, Germany, and University of Giessen Marburg Lung Center (UGMLC); Member of the German Center for Lung Research; **i** Department of General Pediatrics and Neonatology, Saarland University Medical School, Homburg, Germany; **j** University of Cologne, Faculty of Medicine and University Hospital Cologne, Department of Pediatrics, Cologne, Germany; **k** LungenClinic Grosshansdorf GmbH, Grosshansdorf, Germany, and Airway Research Center North (ARCN), Germany; German Center for Lung Research (DZL); **l** Velocity Clinical Research Germany, Ahrensburg, formerly Pulmonary Research Institute at LungenClinic Grosshansdorf, Airway Research Center North (ARCN), German Center for Lung Research (DZL); **m** Research Center Borstel – Medical Clinic, Borstel, Germany, and Airway Research Center North (ARCN), Germany; German Center for Lung Research (DZL); **n** Hannover Medical School, Hannover, Germany, and Biomedical Research in Endstage and Obstructive Lung Disease Hannover (BREATH), Germany; German Center for Lung Research (DZL); **o** Institute of Laboratory Medicine and Pathobiochemistry, Molecular Diagnostics, University of Marburg, Germany,  and University of Gießen, Marburg Lung Center (UGMLC); German Center for Lung Research (DZL); **p** University of Cologne, Faculty of Medicine and University Hospital Cologne, Translational Experimental Pediatrics - Experimental Pulmonology, Department of Pediatric and Adolescent Medicine, Germany; **q**  University of Cologne, Faculty of Medicine and University Hospital Cologne, Center for Molecular Medicine Cologne (CMMC), Germany; **r** Excellence Cluster on Stress Responses in Aging-associated Diseases (CECAD), University of Cologne, Faculty of Medicine and University Hospital Cologne Cologne, Germany; **s**  Institute for Lung Health, University of Giessen and Marburg Lung Centre (UGMLC), Member of the German Centre for Lung Research (DZL), Gießen, Germany; **t** Clinical Respiratory Epidemiology, Dr. von Hauner Children’s Hospital, Ludwig Maximilian University of Munich, Munich, Germany; **u** Division of Epigenetics in Chronic Lung Disease, Priority Area Chronic Lung Diseases, Leibniz Lung Center, Research Center Borstel, Borstel, Germany; **v** Institute for Comparative Microbiome Analysis; Helmholtz Zentrum Munich, Deutsches Forschungszentrum für Gesundheit und Umwelt (GmbH), Munich, Germany; **x** Center of Allergy & Environment (ZAUM), Technical University of Munich and Helmholtz; **y** German Center for Child and Adolescent Health (DZKJ), Dr von Hauner Children's Hospital, LMU Munich, Germany

**Supplementary Figures and Tables**

**
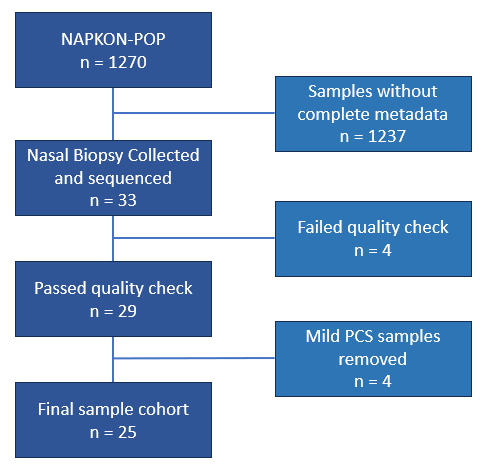
**

**Figure S1: Outline of NAPKON population samples selected for clinical analysis and single-cell RNA-sequencing.**

**
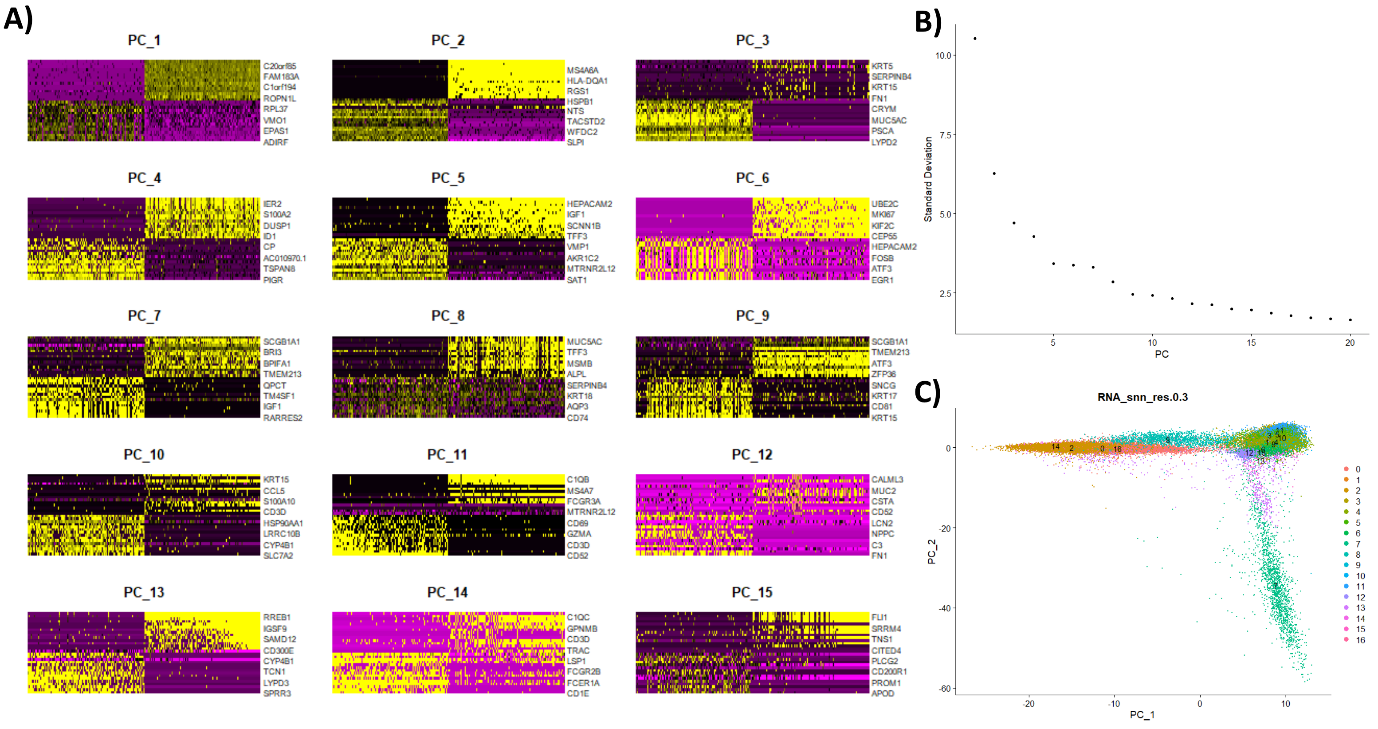
**

**Figure S2: Principal component (PC) analysis of scRNA-seq patient samples**. The dataset's dimensionality was reduced to 100 principal components. Here, we present the analysis of the first 15 principal components of the scRNA-seq patient samples. A) Expression heatmap of genes contributing to the first 15 PCs. B) Elbow plot of the standard deviation (y-axis) captured by each PC (x-axis); n = 25.


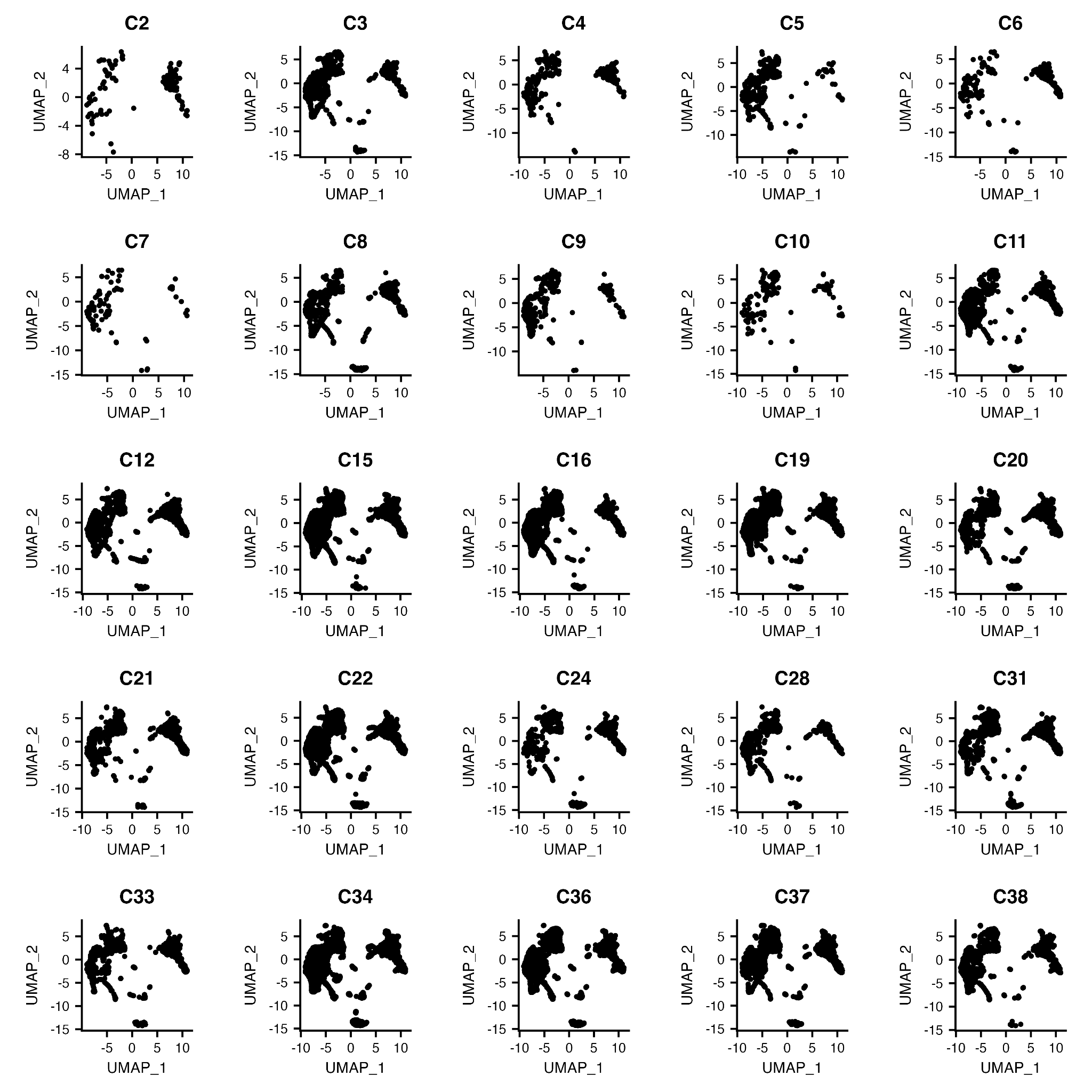


**Figure S3: UMAP visualization of cell populations across 25 samples included in the scRNA-seq analysis.** UMAP visualizations showing the cellular composition of each of the 25 individual samples. Each panel represents a single sample, with cells from that sample depicted in black.


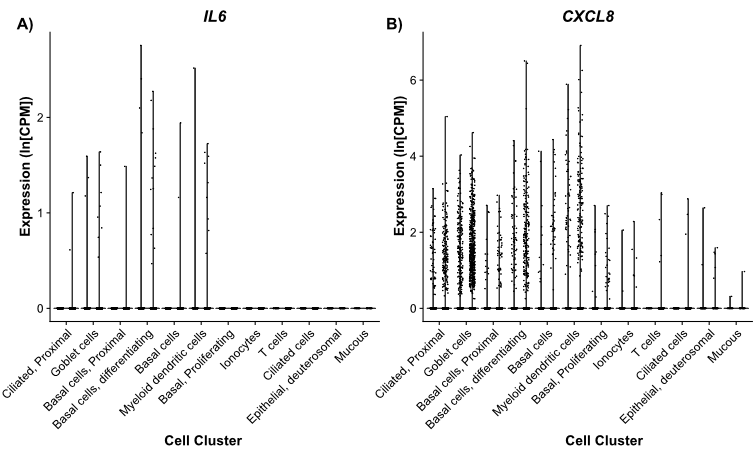


**Figure S4: (A) *IL6* and (B) *CXCL8* gene expression across all cell clusters.** Violin plots of *IL6* and *CXCL8* gene expression measured in ln(counts per million) (y-axis) across the collapsed cell clusters (x-axis). Expression is presented as a grouped comparison with moderate PCS patients presented first and severe PCS patients second. Each individual point represents a single cell; n = 25.

**
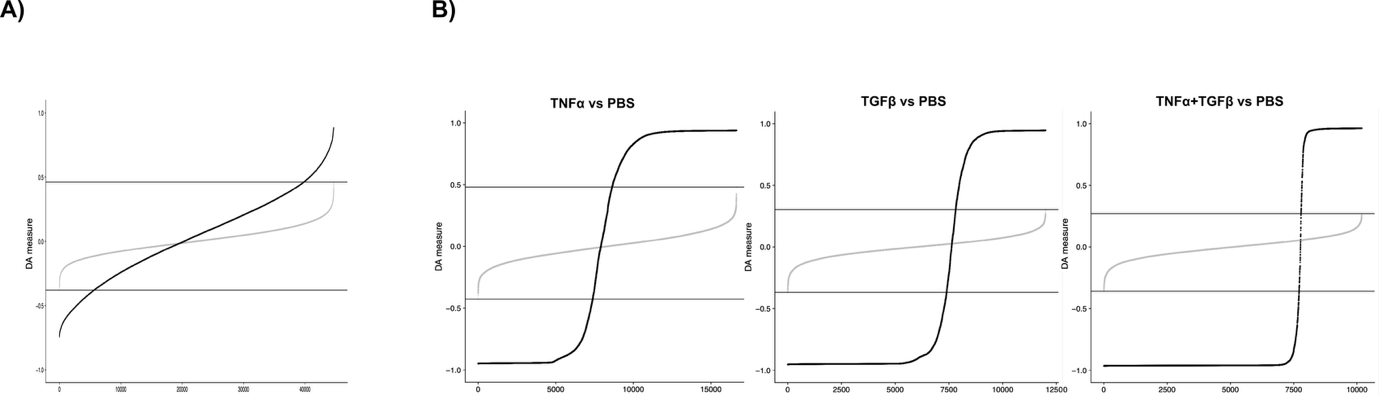
**

**Figure S5. Results of the permutation test for differential abundance (DA) analysis using DAseq**. (A) This part examines the differential abundance between moderate and severe PCS conditions in vivo. The black line represents the observed DA scores, while the gray lines depict the null distribution derived from permuted labels. Deviations from the null distribution indicate significant differential abundance. Horizontal lines mark the DA thresholds used to define strong differential abundance. (B) The results of the permutation test comparing stimulated versus unstimulated ALI samples are shown here. From left to right, the comparisons are TNFα vs PBS, TGFβ vs PBS, and TNFα + TGFβ vs. PBS. For each condition, gray lines illustrate the null distribution from permuted labels, and the black lines indicate the observed DA measures. Horizontal lines indicate the DA thresholds used to establish strong differential abundance, where a significant deviation from the null distribution signifies noteworthy differential abundance under each stimulation condition.


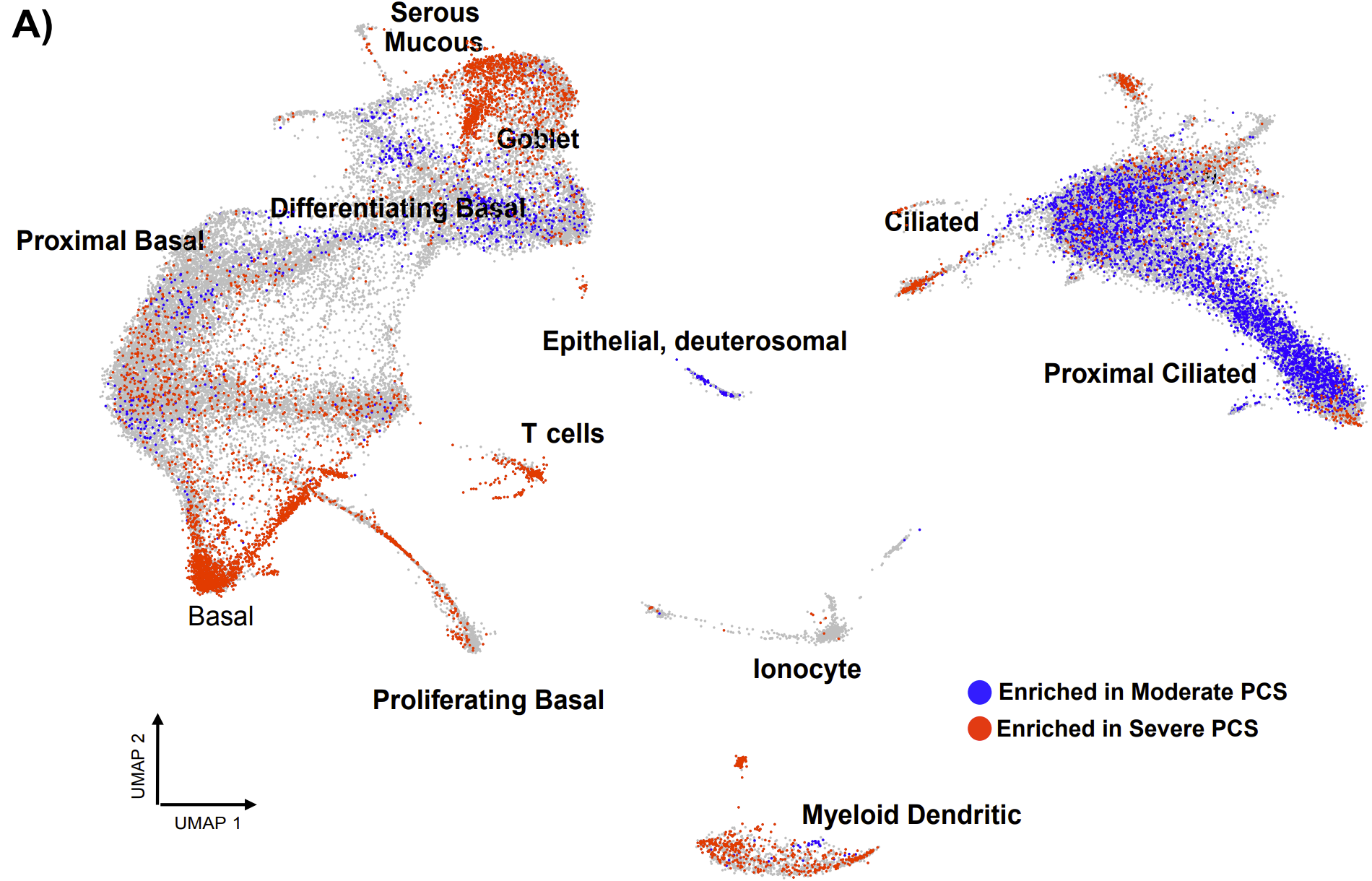


**Figure S6: UMAP representation of differential cell abundance between moderate (blue) and severe (red) PCS.** DA-seq detected differentially abundant cell subpopulations by analysing cells from moderate and severe PCS. Cells coloured blue represent increased abundance in moderate PCS, whilst red cells indicate increased abundance in severe PCS, with grey cells representing equal abundance in moderate and severe PCS. Each point represents an individual cell; n = 25.

**Figure S7: Representation of cellular deconvolution in healthy control patient epithelium.** Healthy control samples were collected as part of the All-Age-Asthma-Cohort^15^. Deconvolution was completed using the DWLS method on bulk RNA-seq samples. Cell frequency is indicated by the y-axis, with unique cell clusters represented by specific colours.

Numbers indicate the proportion each cell type comprises of the total cell population in the sample.

**Figure S8: Gene expression of *MIF* (A), *CD74* (B), *CXCR4* (C) and *CD44* (D) on a UMAP projection of scRNA-seq analysis.** Gene expression is presented as ln(counts per million), with purple representing increased gene expression and yellow representing less expression. Gene expression is shown for patients with moderate (left) and severe (right) PCS; n = 25.
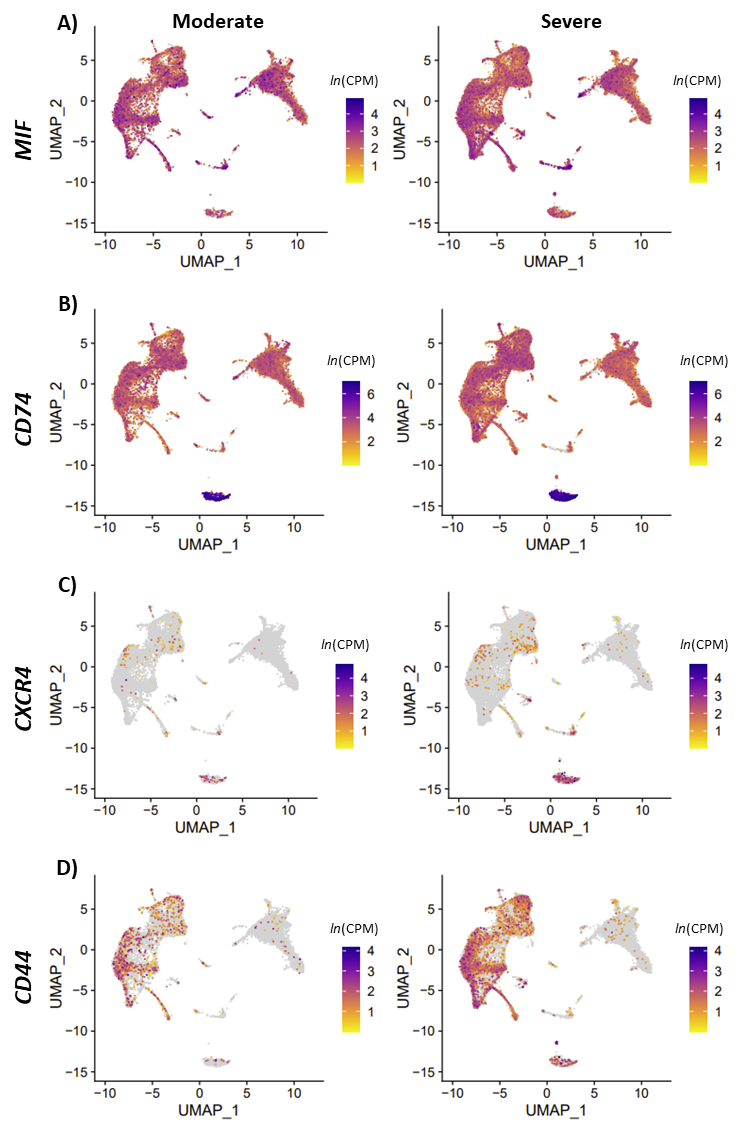


**Figure S9: Representation of significant ligand-receptor pair interactions with basal proliferating cells.** (A) Basal proliferating cells are the receivers of signals from all other cell types. (B) Basal proliferating cells are senders of signals to all other cell types. The x-axis presents all cell types in moderate (pink) and severe (blue) PCS patients. Statistical significance was determined at p < 0.05; n = 25.
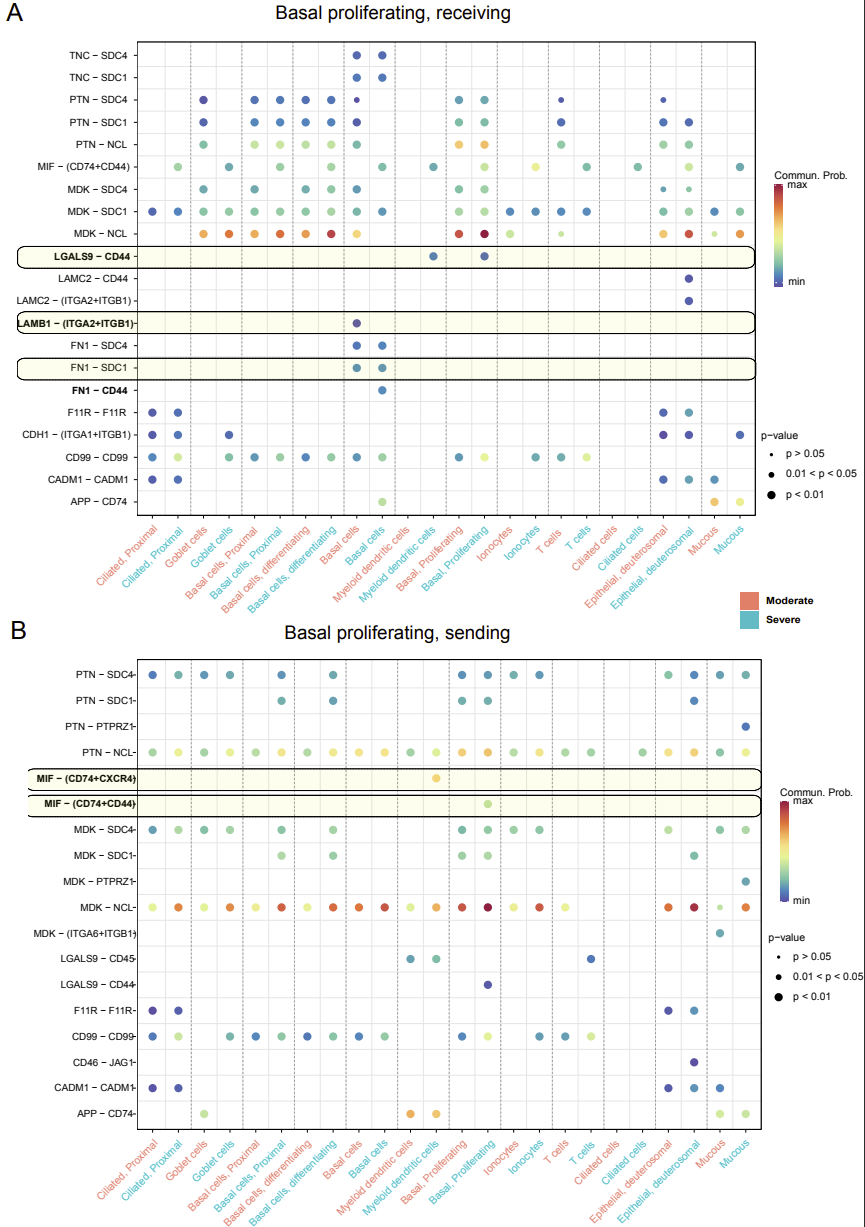


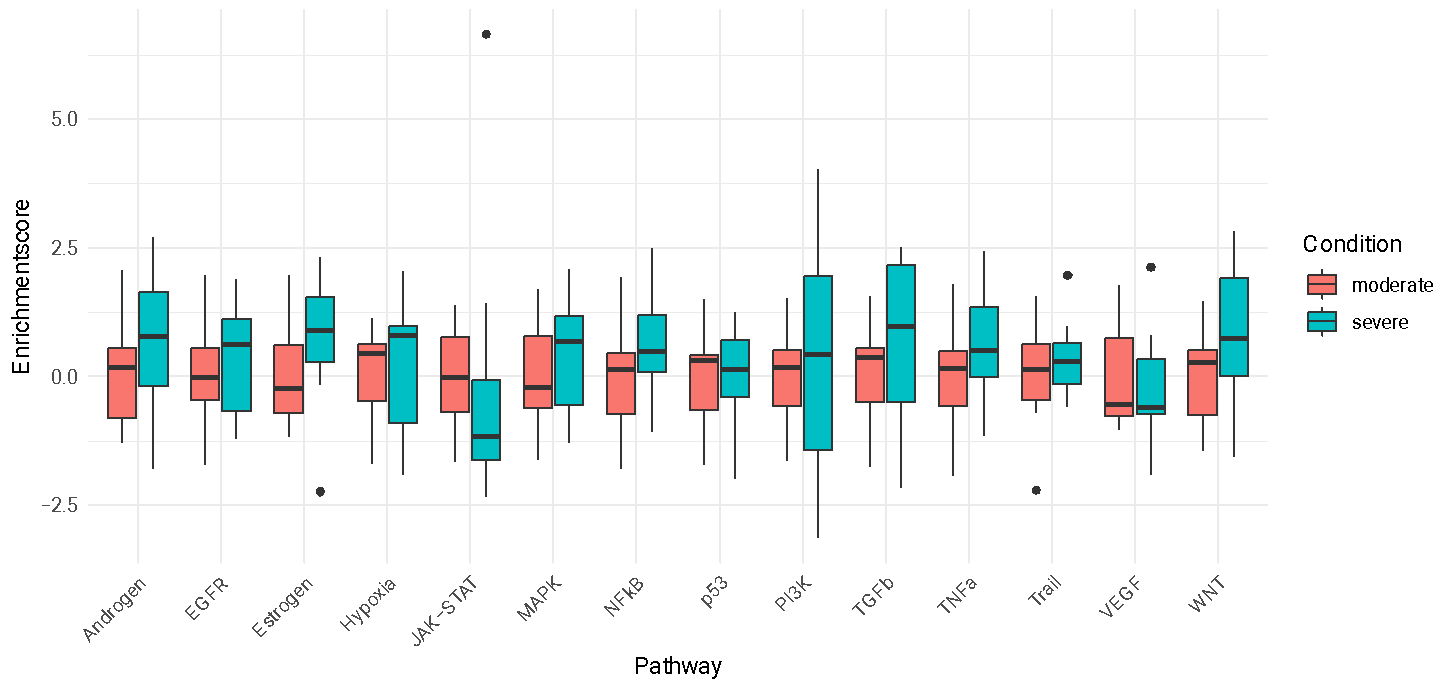


**Figure S10: Boxplot of pathway enrichment scores for moderate and severe patients.** The PROGENy database was used to identify molecular pathways that are differentially regulated between moderate and severe PCS patients^11^. The pathway activity is statistically tested between moderate and severe PCS groups by incorporating a linear model; n = 25.



**Figure S11: Gene expression of *TNFRS1A* (A), *TGFBR1* (B), and *TGFBR2* (C) on a UMAP projection of scRNA-seq analysis.** Gene expression is presented as ln(counts per million), with purple representing increased gene expression and yellow representing less expression. Gene expression is shown for patients with moderate (left) and severe (right) PCS; n = 25.

**
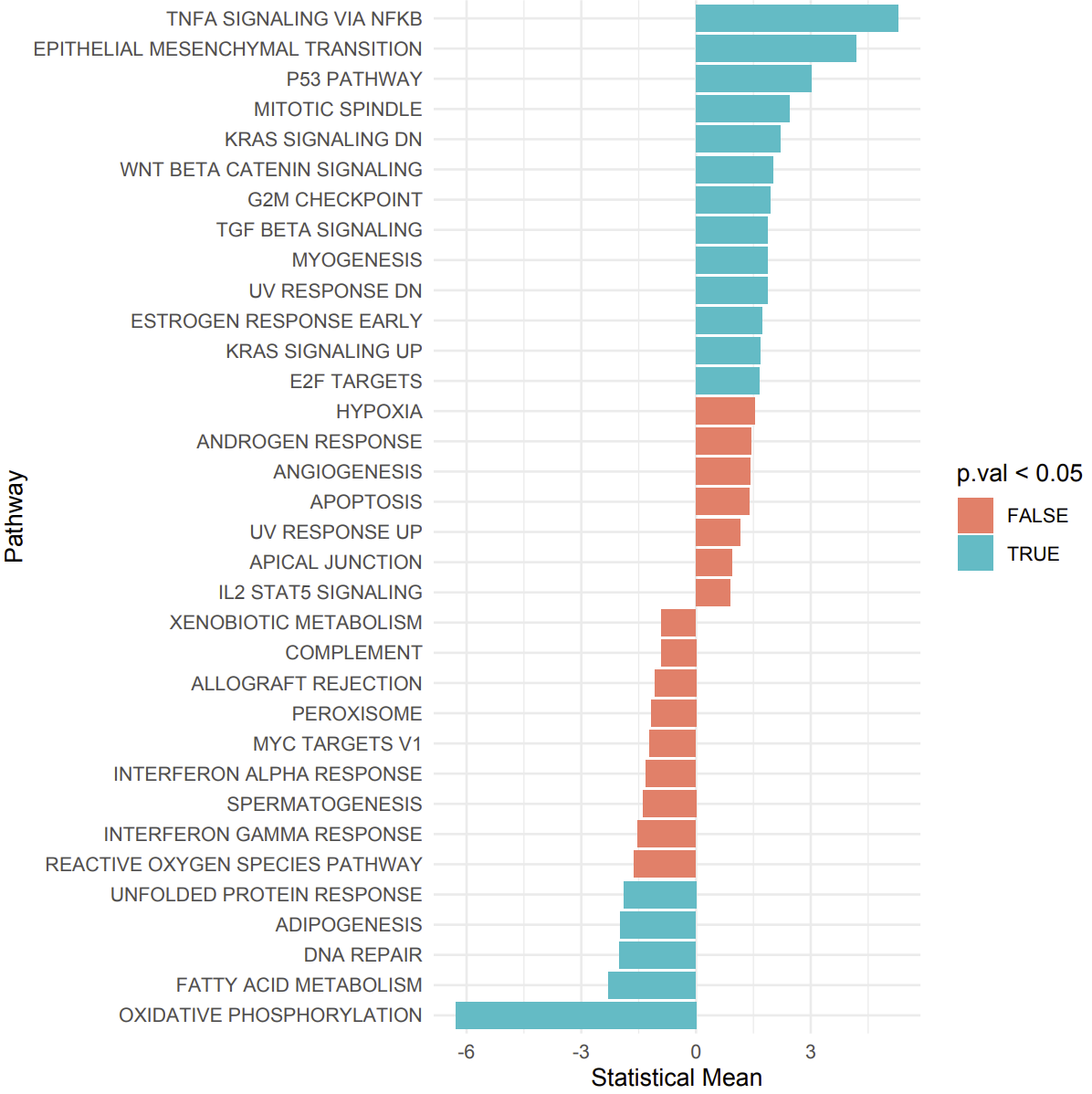
**

**Figure S12: GSEA of pathways enriched between moderate and severe PCS patients.** A positive statistical mean from a rank-based test (x-axis) indicates enrichment in severe PCS, while a negative statistical mean from a rank-based test indicates enrichment in moderate PCS. Statistical significance was determined at p<0.05 indicated in blue, whilst non-significant pathways (p>0.05) are indicated in red; n = 25.

**
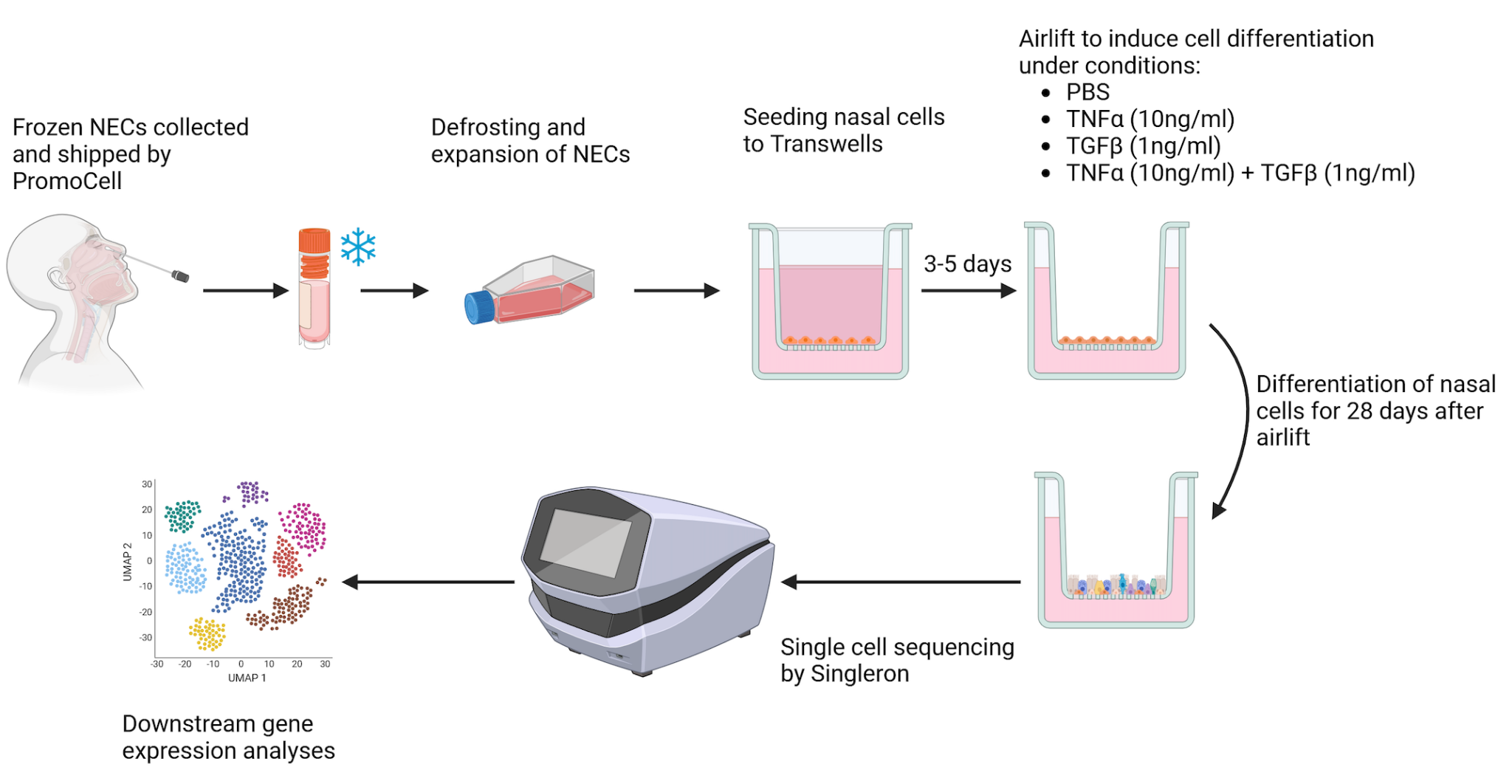
**

**Figure S13: Schematic diagram of air-liquid interface culture (ALI) of primary human nasal epithelial cells**. NECs were exposed to PBS (control), TNFα (10ng/ml), TGFβ (1ng/ml) alone or combined from the day of airlift. For each condition, two biological replicates (n = 2) were subjected to single-cell RNA sequencing (Singleron). *Created in BioRender. Reddy, D. (2025)* [*https://BioRender.com/7r9o3qo*](https://BioRender.com/7r9o3qo)


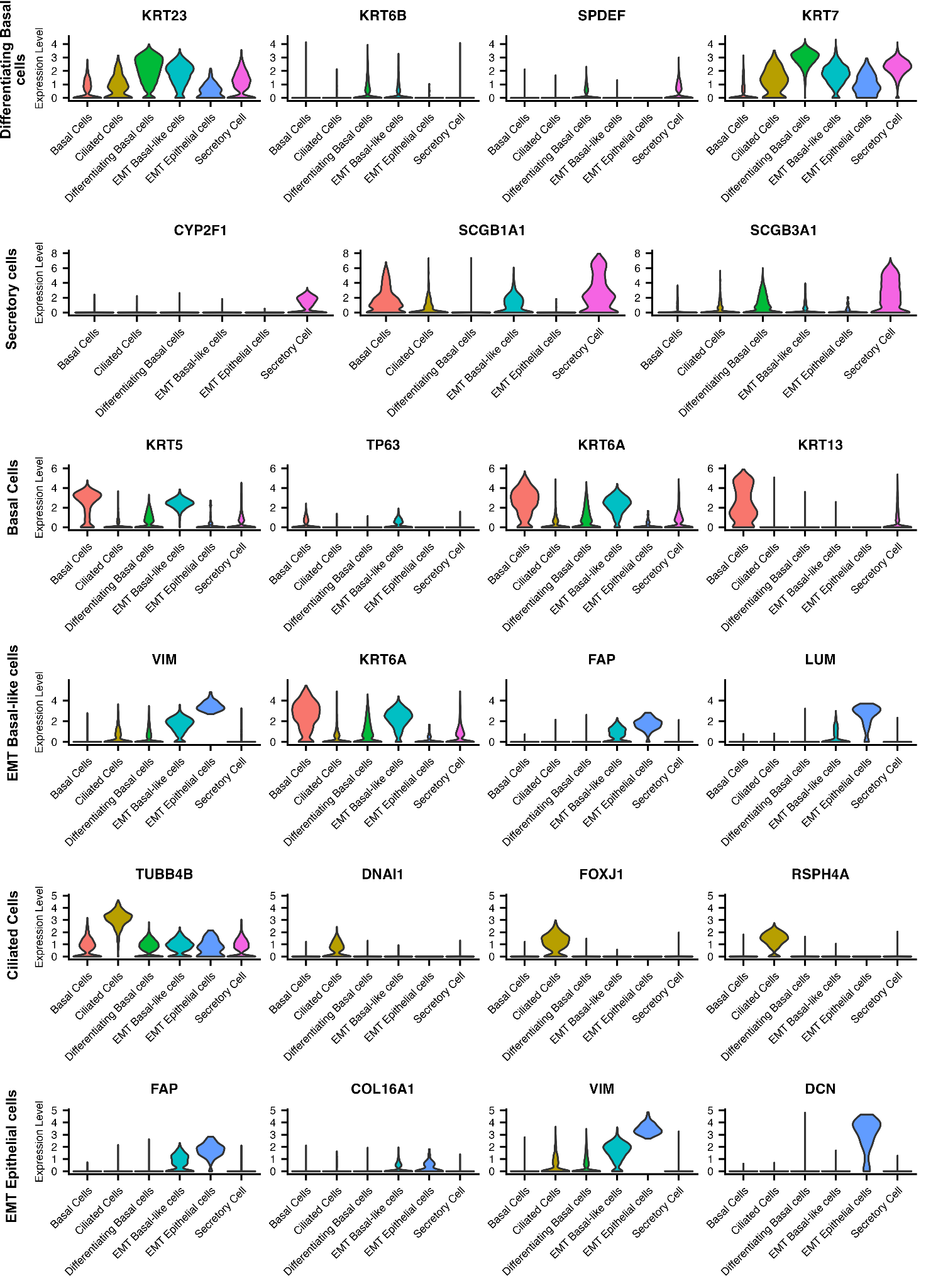


***Figure S14: Expression levels of traditional marker genes characterize distinct cell populations.*** *Violin plots illustrate expression levels of specific marker genes employed to determine and annotate for distinct cell types. Each panel indicates the range of normalized expression values for a specific gene (as depicted above) within the identified cellular clusters (as indicated on the x-axis). The y-axis refers to the level of normalized expression. Panels are clustered to highlight marker genes associated with specific cell lineages.*


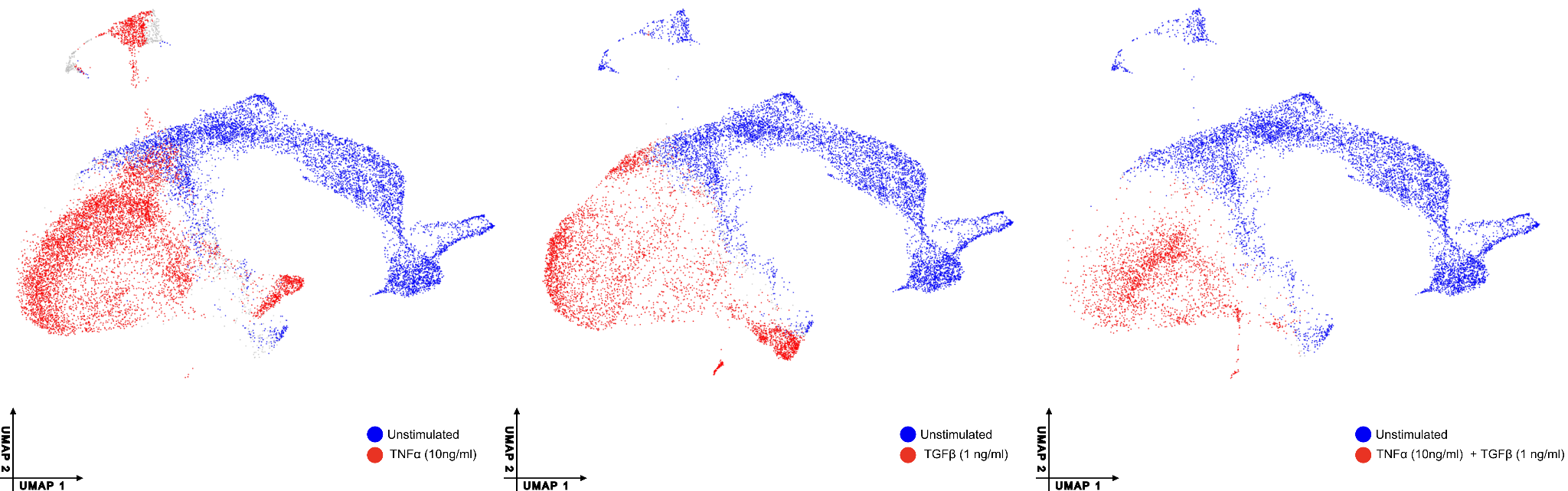
**Figure S15: DASeq analysis across each stimulation condition compared to unstimulated ALI samples.** Uniform Manifold Approximation Projection (UMAP) of differential cell abundance from left to right treatment conditions comparisons PBS vs TNFα (10ng/ml), PBS vs TGFβ (10ng/ml) and TNFα (10ng/ml) + TGFβ (1ng/ml) vs PBS. Blue dots indicate increased cell abundance in unstimulated control samples. Red dots indicate increased cell abundance in the stimulation condition relative to unstimulated controls, n = 2 donors for each condition.


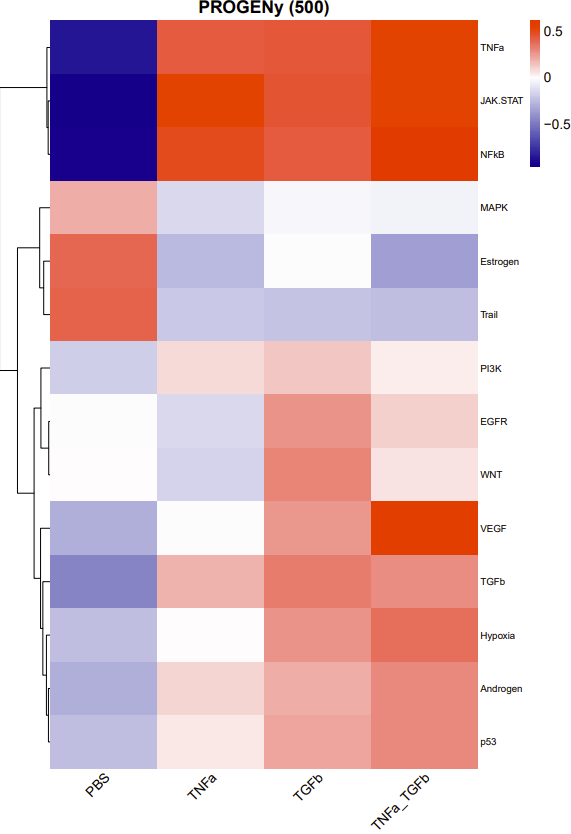


**Figure S16: PROGENy pathway enrichment analysis stratified by ALI differentiation conditions.** The right y-axis states specific activated pathways across experimental conditions. Red = higher enrichment of pathway relative to PBS control conditions; blue = decreased pathway enrichment relative to PBS control conditions, n = 2 donors for each condition.


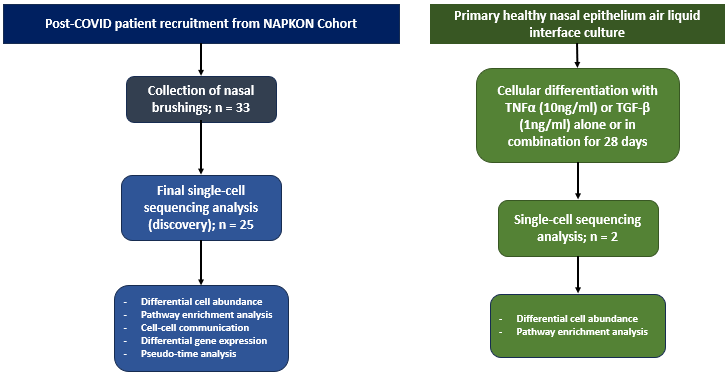

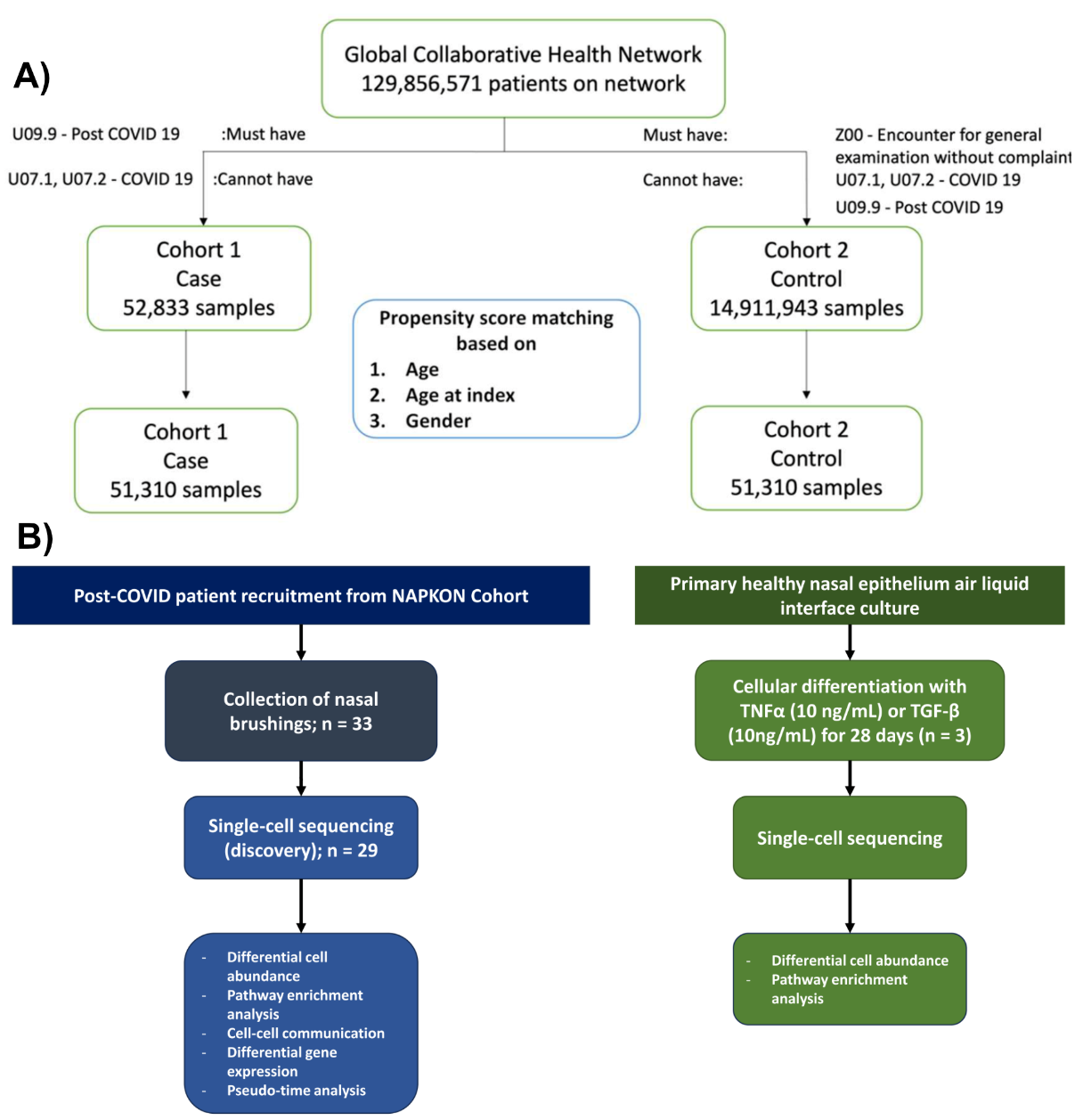


**Figure S17: Schematic overview of the clinical (A), *in vivo* and *in vitro* (B) pipelines.** (A) TriNetX cohort selection and analysis design. (B) NAPKON (blue) and *in vitro* (green) scRNA-seq analysis pipelines.


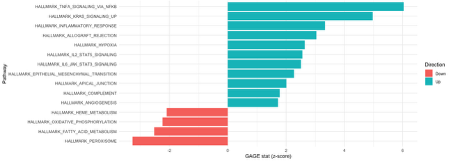


**Figure S18:***:* **GSEA of pathways enriched between moderate and severe PCS patients based on a pseudobulk Difeferantial analysis approach.** We additionally performed a pseudobulk DE analysis using Seurats’s *AggregateExpression* function and DESeq2, followed by GSEA with a Wilcoxon Mann-Whitney test on log fold changes. *A positive statistical mean (x-axis) indicates enrichment in severe PCS, while a negative statistical mean indicates enrichment in moderate PCS. Statistical significance was determined at p<0.05 and respective pathways were shown, n = 25.*

**Supplementary Tables**

**Table S1: Summary of patient clinical data.** Data is presented as the arithmetic mean. The statistical significance was analyzed by a two-tailed Mann-Whitney test for continuous variables (age & PCS score) or by a one-tailed Chi-Square test for categorical frequency data with significance indicated by *p < 0.05 or ****p<0.0001 (moderate PCS vs. severe PCS). SD = standard deviation; ENT = ear, nose, and throat; PCS = post-COVID syndrome.

**
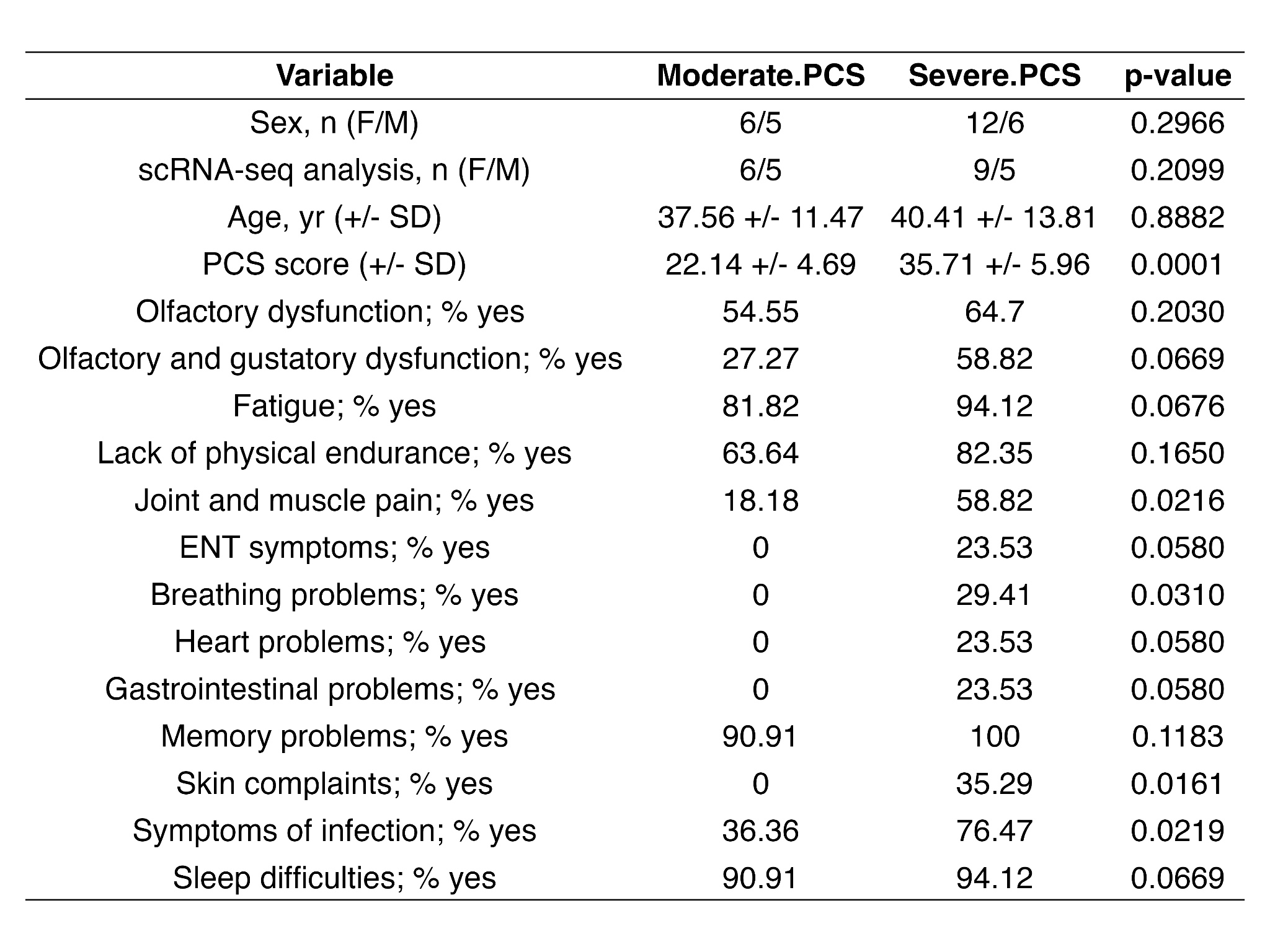
**

**Table S2: Differentially gene expression and canonical markers for cluster annotation.** Due to its size and inclusion of multiple sheets, the complete table is provided as a separate supplementary file as Source data (Supplementary Table S2 - DEGs.xlsx). Differentially expressed genes were identified using the Seurat package with the MAST test. The MAST framework implements a hurdle model fit to log-normalized expression values, and two-sided hypothesis testing was applied. P values were adjusted for multiple comparisons using the bonferroni correction method. The table reports for each cell type (separated by different sheets) each gene, the log fold-change, average expression, percent expression in the cluster of interest versus all other clusters, raw P values, and adjusted P values.

**Table S3: Quantification of cell abundance differences between moderate and severe PCS.** The scProportionTest quantifies differences in cell abundance between clusters from moderate and severe samples. A two-sided permutation test (n = 1000) was performed to calculate statistical p-values for each cluster, and confidence intervals for the magnitude of differences were estimated via bootstrapping. Obs_log2FD = observed log2 fold difference; pval = p-value.

**
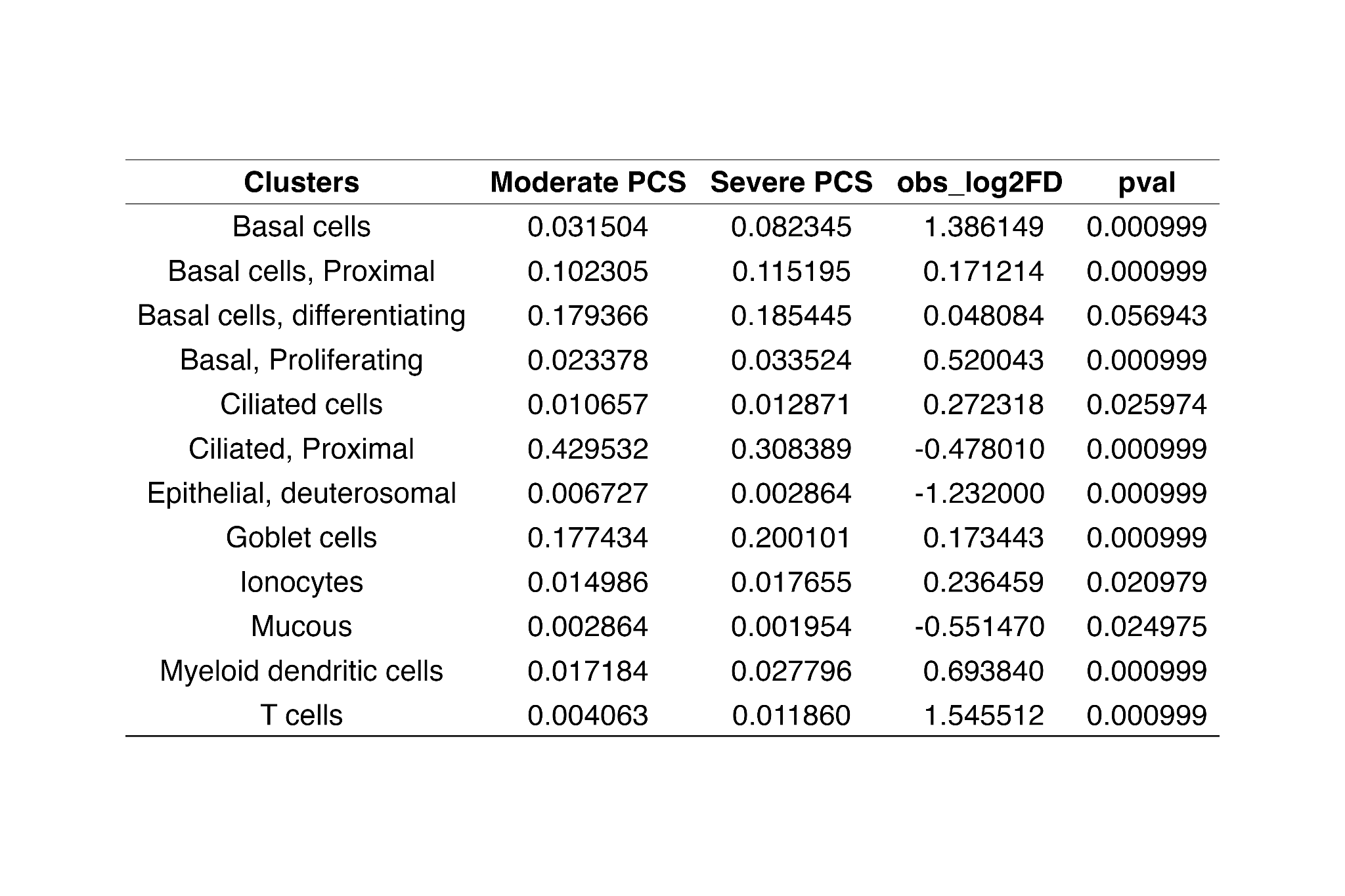
**

**Table S4: Correlation of post-COVID syndrome and different respiratory diseases.** Hazard ratios were estimated using Cox proportional hazards models, and odds ratios were calculated with logistic regression models on the TriNetX platform. Prior logistic regression model, propensity score matching were used for confounding control. All tests were two-sided, and no adjustments were made for multiple comparisons. The results include hazard ratios, odds ratios, 95% confidence intervals, and exact P values. Patient cohort sizes varied slightly across analyses, ranging from 49,935 to 51,310 cases and about 15,797,934 control cohort samples. This variation occurred because of the real-time, federated nature of the database.

**
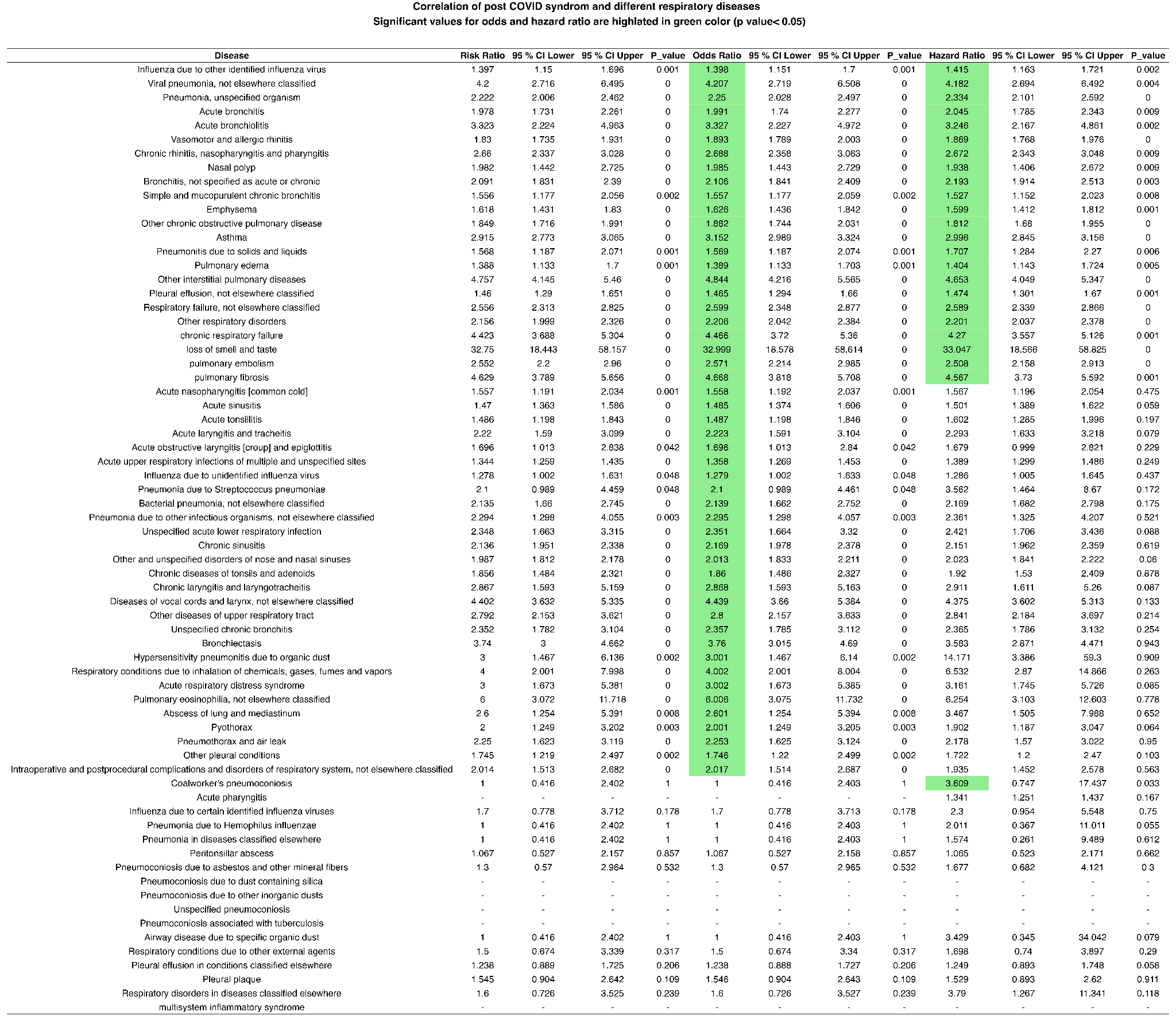
**

**Table S5:** **Quantification of cell abundance differences between NECs under different stimulation conditions in an air-liquid interface (ALI) model**. The scProportionTest was used to assess differences in cell abundance between clusters across three comparisons: TNFα vs PBS, TGFβ vs PBS, and TNFα+TGFβ vs PBS. A two-sided permutation test (n = 1000) was performed to calculate statistical p-values for each cluster, and confidence intervals for the magnitude of differences were estimated via bootstrapping. Obs_log2FD = observed log2 fold difference; pval = p-value; FDR = false discovery rate.


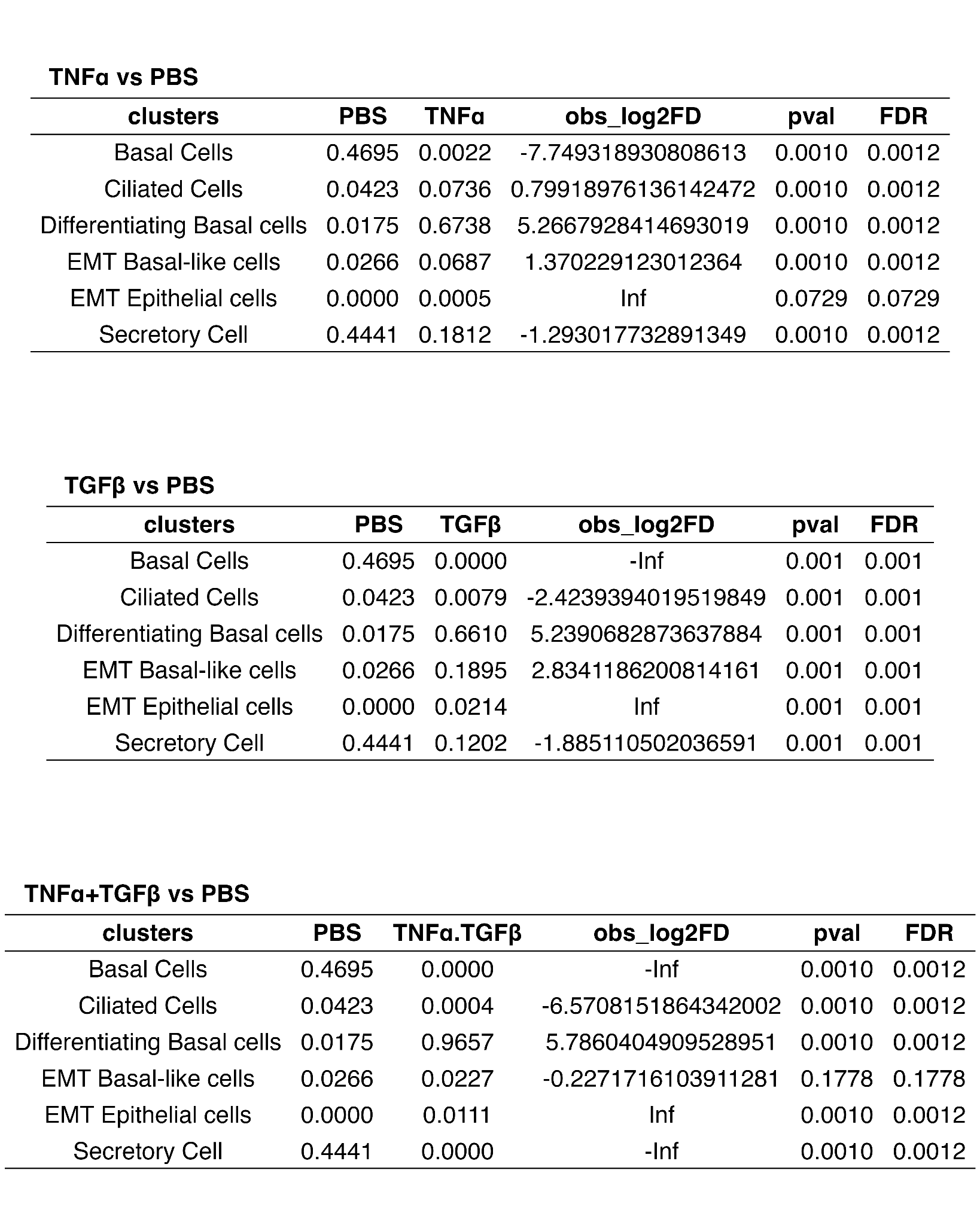


**References**

1. McGinnis CS, Murrow LM, Gartner ZJ. DoubletFinder: doublet detection in single-cell RNA sequencing data using artificial nearest neighbors. *Cell systems* **8**, 329-337. e324 (2019).

2. Mercer TR*, et al.* The human mitochondrial transcriptome. *Cell* **146**, 645-658 (2011).

3. Korsunsky I*, et al.* Fast, sensitive and accurate integration of single-cell data with Harmony. *Nature methods* **16**, 1289-1296 (2019).

4. Tran HTN*, et al.* A benchmark of batch-effect correction methods for single-cell RNA sequencing data. *Genome biology* **21**, 1-32 (2020).

5. Clarke ZA*, et al.* Tutorial: guidelines for annotating single-cell transcriptomic maps using automated and manual methods. *Nature protocols* **16**, 2749-2764 (2021).

6. Khan M*, et al.* Visualizing in deceased COVID-19 patients how SARS-CoV-2 attacks the respiratory and olfactory mucosae but spares the olfactory bulb. *Cell* **184**, 5932-5949. e5915 (2021).

7. Holbrook EH, Wu E, Curry WT, Lin DT, Schwob JE. Immunohistochemical characterization of human olfactory tissue. *The Laryngoscope* **121**, 1687-1701 (2011).

8. Miller SA*, et al.* LSD1 and aberrant DNA methylation mediate persistence of enteroendocrine progenitors that support BRAF-mutant colorectal cancer. *Cancer research* **81**, 3791-3805 (2021).

9. Palchuk MB*, et al.* A global federated real-world data and analytics platform for research. *JAMIA open* **6**, ooad035 (2023).

10. Jin S*, et al.* Inference and analysis of cell-cell communication using CellChat. *Nature communications* **12**, 1088 (2021).

11. Schubert M*, et al.* Perturbation-response genes reveal signaling footprints in cancer gene expression. *Nature communications* **9**, 20 (2018).

12. Finak, G., McDavid, A., Yajima, M. *et al.* MAST: a flexible statistical framework for assessing transcriptional changes and characterizing heterogeneity in single-cell RNA sequencing data. *Genome Biol* 16, 278 (2015).

13. Nguyen, H.C.T., Baik, B., Yoon, S. *et al.* Benchmarking integration of single-cell differential expression. *Nat Commun* 14, 1570 (2023).

14. Luo W, Friedman MS, Shedden K, Hankenson KD, Woolf PJ. GAGE: generally applicable gene set enrichment for pathway analysis. *BMC bioinformatics* **10**, 1-17 (2009).

15. Trapnell C*, et al.* The dynamics and regulators of cell fate decisions are revealed by pseudotemporal ordering of single cells. *Nature biotechnology* **32**, 381-386 (2014).

16. Tsoucas D, Dong R, Chen H, Zhu Q, Guo G, Yuan G-C. Accurate estimation of cell-type composition from gene expression data. *Nature communications* **10**, 2975 (2019).
