# Supplementary material for: scRNA-seq reveals persistent aberrant differentiation of nasal epithelium driven by TNFα and TGFβ in post-COVID syndrome
Source: Nat Commun. 2025 Oct 28;16:9494. doi: 10.1038/s41467-025-64778-0 (PMC12569062; doi:10.1038/s41467-025-64778-0)
Supplement: Supplementary file 2 — Reporting Summary [file 41467_2025_64778_MOESM2_ESM.pdf]

nature portfolio

Corresponding author(s): Anke Fähnrich

Last updated by author(s): 17.09.2025

# Reporting Summary

Nature Portfolio wishes to improve the reproducibility of the work that we publish. This form provides structure for consistency and transparency in reporting. For further information on Nature Portfolio policies, see our Editorial Policies and the Editorial Policy Checklist.

## Statistics

For all statistical analyses, confirm that the following items are present in the figure legend, table legend, main text, or Methods section.

| n/a | Confirmed | |
|---|---|---|
| ☐ | ☒ | The exact sample size (*n*) for each experimental group/condition, given as a discrete number and unit of measurement |
| ☐ | ☒ | A statement on whether measurements were taken from distinct samples or whether the same sample was measured repeatedly |
| ☐ | ☒ | The statistical test(s) used AND whether they are one- or two-sided<br>*Only common tests should be described solely by name; describe more complex techniques in the Methods section.* |
| ☐ | ☒ | A description of all covariates tested |
| ☐ | ☒ | A description of any assumptions or corrections, such as tests of normality and adjustment for multiple comparisons |
| ☐ | ☒ | A full description of the statistical parameters including central tendency (e.g. means) or other basic estimates (e.g. regression coefficient) AND variation (e.g. standard deviation) or associated estimates of uncertainty (e.g. confidence intervals) |
| ☐ | ☒ | For null hypothesis testing, the test statistic (e.g. *F*, *t*, *r*) with confidence intervals, effect sizes, degrees of freedom and *P* value noted<br>*Give P values as exact values whenever suitable.* |
| ☐ | ☒ | For Bayesian analysis, information on the choice of priors and Markov chain Monte Carlo settings |
| ☐ | ☒ | For hierarchical and complex designs, identification of the appropriate level for tests and full reporting of outcomes |
| ☒ | ☐ | Estimates of effect sizes (e.g. Cohen's *d*, Pearson's *r*), indicating how they were calculated |

*Our web collection on statistics for biologists contains articles on many of the points above.*

## Software and code

Policy information about availability of computer code

| | |
|---|---|
| Data collection | No custom or commercial code was specifially used for the collection of  raw sequencing data. |
| Data analysis | python (v.3.8.17), numpy (v.1.24.3), kallisto (v.0.46.1), bustools  (v.0.46.1), R (v.4.2.1), DropletUtils (v.1.8.0), doubletFinder (v.2.0),'Seurat' (v.4.3),  Harmony, STAR (v.2.7.3), scProportionTest (v.0.0.0.9000), DAseq (v.1.0.0), CellChat (v.1), PROGENy (v.1.28.0), gage (v. 2.52), Monocle (v.2.6.4),  DWLS (v.0.1.0) |

For manuscripts utilizing custom algorithms or software that are central to the research but not yet described in published literature, software must be made available to editors and reviewers. We strongly encourage code deposition in a community repository (e.g. GitHub). See the Nature Portfolio guidelines for submitting code & software for further information.

## Data

Policy information about availability of data

All manuscripts must include a data availability statement. This statement should provide the following information, where applicable:

- Accession codes, unique identifiers, or web links for publicly available datasets
- A description of any restrictions on data availability
- For clinical datasets or third party data, please ensure that the statement adheres to our policy

The ALLIANCE data used in this study are sensitive due to individual patient-level data it will be available upon reasonable request. scRNA-seq data, for the ALI culture generated in this study have been deposited in the GEO database under accession number: GSE299529 (https://www.ncbi.nlm.nih.gov/geo/query/acc.cgi?acc=GSE299529)

## Research involving human participants, their data, or biological material

Policy information about studies with human participants or human data. See also policy information about sex, gender (identity/presentation), and sexual orientation and race, ethnicity and racism.

| | |
|---|---|
| Reporting on sex and gender | In April 2020, the German government launched the Network University Medicine (NUM) to coordinate and support COVID-19-related research efforts at the national level. As part of this initiative, The National Pandemic Cohort Network, NAKON was established, including the population-based cohort platform NAPKON-POP. Within this framework, the COVID study was initiated as a prospective, cohort study investigating the long-term health consequences of SARS-CoV-2 infection.<br><br>For the present analysis, we selected a subset of 33 patients from the NAKON cohort (n = 1,270) for whom complete metadata were available. The selected patient groups were of comparable age, and no significant sex differences were observed between the moderate and severe post-COVID syndrome (PCS) groups. The findings are not limited to a single sex or gender. Sex was defined based on self-report. The experiment was designed to be matched on the basis of biological sex in sample collection for downstream scRNA-sequencing analysis. Due to samples quality some samples could not be analysed. A third group of interest was removed from downstream analysis as only male samples passed quality checks and would have therefore introduced a strong sex bias. |
| Reporting on race, ethnicity, or other socially relevant groupings | No socially relevant categorization variables, such as socioeconomic status, race/ethnicity, or educational background, were included or analyzed in the present study. As stated, patients were recruited as part of the German NAPKON program resulting all patients orginating from similar German ethnic origin. |
| Population characteristics | Covariate population characteristics are detailed in Supplementary Table S1, which summarizes the clinical data of the study participan. Data are presented as arithmetic means with standard devaiations (SD). Statistical comparisions were performed using the Mann-Whitney U test for continuous variables (e.g., age and PCS score) and the Chi-square test for categorical variables. Statistical significance is denoted as *p < 0.05 or ****p < 0.0001 (moderate PCS vs. severe PCS). |
| Recruitment | The criteria for patient inclusion were (i) polymerase chain reaction confirmed SARS-CoV-2 infection and persistence of COVID-19 symptoms for more than three months, (ii) post-acute symptom development, (iii) a worsening of pre-existing comorbidities and (iv) written and informed consent before biopsy collection, aligning with ethical approval. |
| Ethics oversight | The study was approved by the ethics committee under approval number D537/20, as part of the NAKON-POP cohort, which is conducted within the framework of the German COVID-19 Research Network of University Medicine (National Pandemic Cohort Network - NAKON). |

Note that full information on the approval of the study protocol must also be provided in the manuscript.

# Field-specific reporting

Please select the one below that is the best fit for your research. If you are not sure, read the appropriate sections before making your selection.

☒ Life sciences ☐ Behavioural & social sciences ☐ Ecological, evolutionary & environmental sciences

For a reference copy of the document with all sections, see nature.com/documents/nr-reporting-summary-flat.pdf

# Life sciences study design

All studies must disclose on these points even when the disclosure is negative.

| | |
|---|---|
| Sample size | At the time this study was initiated, single-cell data were predominantly obtained either post-mortem from lung tissue, from epithelial, lining fluid, or from peripheral blood mononuclear cells (PBMCs) in hospitalized patients. However, implementing single-cell analysis in routine clinical settings remains challenging due to several factors: the need for immediate sample processing, the limited availability of fresh tissue, and the lack of large, balanced reference datasets. Consequently, single-cell approaches have not yet been widely adopted in clinical diagnostics, and their potential for patient stratification remains to be established.<br><br>Given these technical and logistical limitations, only a few studies have successfully employed single-cell sequencing in a clinical context. In light of the complexity and resource-intensiveness of this methodology, a sample size of 33 patients was selected as feasible and representative for the exploratory nature of the study. Although no formal power calculation was conducted, based on existing datasets and effect size estimates this sample size is sufficient to yield meaningful insights into cellular heterogeneity in post-COVID syndrome. |
| Data exclusions | A total of 33 patients were initially selected as a feasible and representative sample size, appropriate for the exploratory nature of this single-cell study. Four samples failed to meet initial quality control criteria after sequencing and were excluded. The remaining 29 samples yielded a total of 56,624 high quality cells, each characterized by less than 25% mitochondrial gene content and detection of over 200 genes per cell. The subgroup of patients with mild post-COVID syndrome (PCS) consisted of only four male individuals, resulting in statistically small and sex-imbalanced cohort. To ensure robust and interpretable group comparisons, this subgroup was excluded from downstream analysis. |
| Replication | To investigate the potential causal role of prominent cytokines (TNF-α and TGF-β) in the reduction of ciliated epithelial cells, primary basal nasal epithelial cells (NECs) were cultured in an air-liquid interface (ALI) model and stimulated with TFN and TGF, either individually or in combination (see experimental workflow in Supplementary Figure S13). Each condition was tested using NECs derived from two independent |

donors (n=2 per condition).

Randomization

COVIDOM participants were recruited in catchment areas around Kiel (Northern Germany) and Wuerzburg (Southern Germany), and in the Neukölln district of Berlin (Eastern Germany).
Main inclusion criteria were (i) a polymerase chain reaction confirmed SARS-CoV-2 infection and (ii) a period of at least 6 months between the infection and the visit to the COVIDOM study site. Other
inclusion criteria were > 18 years of age and written informed concent. Key exclusion criterion were an acute reinfection with SARS-CoV-2. Eligible individuals were identified through local public health authorities so as to address an unbiased subpopulation regarding age, sex, hospitalization.and media literacy . Before the study site visit, participants received a questionnaire to complete at home or online (Bahmer et al eClinicalMedicine, Volume 51, 101549, Supplementary material, page 4). The questionnaire, which was based on previous experience with local and national epidemiological projects, covered basic demographic characteristics, general lifestyle, course of disease, circumstances of acute SARS-CoV-2 infection, pre comorbidities, healtcare utilization, and symptom persistence

Blinding

The investigators were blinded in analysis.

# Reporting for specific materials, systems and methods

We require information from authors about some types of materials, experimental systems and methods used in many studies. Here, indicate whether each material, system or method listed is relevant to your study. If you are not sure if a list item applies to your research, read the appropriate section before selecting a response.

## Materials & experimental systems

| n/a | Involved in the study |
|---|---|
| ☐ | ☐ Antibodies |
| ☐ | ☒ Eukaryotic cell lines |
| ☐ | ☐ Palaeontology and archaeology |
| ☐ | ☐ Animals and other organisms |
| ☐ | ☐ Clinical data |
| ☐ | ☐ Dual use research of concern |
| ☐ | ☐ Plants |

## Methods

| n/a | Involved in the study |
|---|---|
| ☒ | ☐ ChIP-seq |
| ☒ | ☐ Flow cytometry |
| ☒ | ☐ MRI-based neuroimaging |

## Antibodies

Antibodies used

*Describe all antibodies used in the study; as applicable, provide supplier name, catalog number, clone name, and lot number.*

Validation

*Describe the validation of each primary antibody for the species and application, noting any validation statements on the manufacturer's website, relevant citations, antibody profiles in online databases, or data provided in the manuscript.*

## Eukaryotic cell lines

Policy information about cell lines and Sex and Gender in Research

Cell line source(s)

Human primary nasal epithelial cells (NEQs) were obtained from PromoCell (Catalog #C-12620, PromoCell GmbH, Heidelberg, Germany). PromoCell isolates these cells from healthy human donors. For this study, cells were derived from two independent donors, one male and one female, as specified by the supplier at the time of purchase.

Authentication

The primary cells were purchased from PromoCell and authenticated by the provider.

Mycoplasma contamination

Primary human nasal epithelial cells (PromoCell, #C-12620) were tested by the supplier for mycoplasma contamination using PCR-based methods. A Certificate of analysis confirming the absence of contamination was provided by the manufactu.rer

Commonly misidentified lines
(See ICLAC register)

*Name any commonly misidentified cell lines used in the study and provide a rationale for their use.*

## Palaeontology and Archaeology

Specimen provenance

*Provide provenance information for specimens and describe permits that were obtained for the work (including the name of the issuing authority, the date of issue, and any identifying information). Permits should encompass collection and, where applicable, export.*

Specimen deposition

*Indicate where the specimens have been deposited to permit free access by other researchers.*

Dating methods

*If new dates are provided, describe how they were obtained (e.g. collection, storage, sample pretreatment and measurement), where they were obtained (i.e. lab name), the calibration program and the protocol for quality assurance OR state that no new dates are*

provided.

☐ Tick this box to confirm that the raw and calibrated dates are available in the paper or in Supplementary Information.

Ethics oversight — *Identify the organization(s) that approved or provided guidance on the study protocol, OR state that no ethical approval or guidance was required and explain why not.*

Note that full information on the approval of the study protocol must also be provided in the manuscript.

# Animals and other research organisms

Policy information about studies involving animals; ARRIVE guidelines recommended for reporting animal research, and Sex and Gender in Research

Laboratory animals — *For laboratory animals, report species, strain and age OR state that the study did not involve laboratory animals.*

Wild animals — *Provide details on animals observed in or captured in the field; report species and age where possible. Describe how animals were caught and transported and what happened to captive animals after the study (if killed, explain why and describe method; if released, say where and when) OR state that the study did not involve wild animals.*

Reporting on sex — *Indicate if findings apply to only one sex; describe whether sex was considered in study design, methods used for assigning sex. Provide data disaggregated for sex where this information has been collected in the source data as appropriate; provide overall numbers in this Reporting Summary. Please state if this information has not been collected. Report sex-based analyses where performed, justify reasons for lack of sex-based analysis.*

Field-collected samples — *For laboratory work with field-collected samples, describe all relevant parameters such as housing, maintenance, temperature, photoperiod and end-of-experiment protocol OR state that the study did not involve samples collected from the field.*

Ethics oversight — *Identify the organization(s) that approved or provided guidance on the study protocol, OR state that no ethical approval or guidance was required and explain why not.*

Note that full information on the approval of the study protocol must also be provided in the manuscript.

# Clinical data

Policy information about clinical studies

All manuscripts should comply with the ICMJE guidelines for publication of clinical research and a completed CONSORT checklist must be included with all submissions.

Clinical trial registration — *Provide the trial registration number from ClinicalTrials.gov or an equivalent agency.*

Study protocol — *Note where the full trial protocol can be accessed OR if not available, explain why.*

Data collection — *Describe the settings and locales of data collection, noting the time periods of recruitment and data collection.*

Outcomes — *Describe how you pre-defined primary and secondary outcome measures and how you assessed these measures.*

# Dual use research of concern

Policy information about dual use research of concern

## Hazards

Could the accidental, deliberate or reckless misuse of agents or technologies generated in the work, or the application of information presented in the manuscript, pose a threat to:

| No | Yes | |
|---|---|---|
| ☒ | ☐ | Public health |
| ☒ | ☐ | National security |
| ☒ | ☐ | Crops and/or livestock |
| ☒ | ☐ | Ecosystems |
| ☒ | ☐ | Any other significant area |

## Experiments of concern

Does the work involve any of these experiments of concern:

| No | Yes | |
|---|---|---|
| ☒ | ☐ | Demonstrate how to render a vaccine ineffective |
| ☒ | ☐ | Confer resistance to therapeutically useful antibiotics or antiviral agents |
| ☒ | ☐ | Enhance the virulence of a pathogen or render a nonpathogen virulent |
| ☒ | ☐ | Increase transmissibility of a pathogen |
| ☒ | ☐ | Alter the host range of a pathogen |
| ☒ | ☐ | Enable evasion of diagnostic/detection modalities |
| ☒ | ☐ | Enable the weaponization of a biological agent or toxin |
| ☒ | ☐ | Any other potentially harmful combination of experiments and agents |

# Plants

| | |
|---|---|
| Seed stocks | *Report on the source of all seed stocks or other plant material used. If applicable, state the seed stock centre and catalogue number. If plant specimens were collected from the field, describe the collection location, date and sampling procedures.* |
| Novel plant genotypes | *Describe the methods by which all novel plant genotypes were produced. This includes those generated by transgenic approaches, gene editing, chemical/radiation-based mutagenesis and hybridization. For transgenic lines, describe the transformation method, the number of independent lines analyzed and the generation upon which experiments were performed. For gene-edited lines, describe the editor used, the endogenous sequence targeted for editing, the targeting guide RNA sequence (if applicable) and how the editor was applied.* |
| Authentication | *Describe any authentication procedures for each seed stock used or novel genotype generated. Describe any experiments used to assess the effect of a mutation and, where applicable, how potential secondary effects (e.g. second site T-DNA insertions, mosiacism, off-target gene editing) were examined.* |
